# Lubiprostone stimulates small intestinal mucin release

**DOI:** 10.1186/1471-230X-12-156

**Published:** 2012-11-06

**Authors:** Robert C De Lisle

**Affiliations:** 1 Anatomy and Cell Biology, University of Kansas School of Medicine, Kansas City, KS, 66160, USA

**Keywords:** Intestine, Lubiprostone, Mucin, Prostaglandin

## Abstract

**Background:**

Lubiprostone is a synthetic bicyclic fatty acid derivative of prostaglandin E1 (PGE_1_) used for chronic constipation. The best known action of lubiprostone is simulation of Cl^-^ dependent fluid secretion. In a mouse model of the genetic disease cystic fibrosis, we previously showed that in vivo administration of lubiprostone resulted in greater mucus accumulation in the small intestine. The aim of this study was to directly test whether lubiprostone stimulates intestinal mucin release.

**Methods:**

Mucin release was measured by mounting segments (4-5 cm) of mouse proximal-mid small intestine in an organ bath, allowing access to the perfusate (luminal) and the bath (serosal) solutions. Nifedipine (10^-6^ M) and indomethacin (10^-5^ M) were included in all solutions to inhibit smooth muscle activity and endogenous prostaglandin production, respectively. The tissue was equilibrated under flow for 30 min, using the perfusate collected during the final 10 min of the equilibration period to measure unstimulated release rate. Stimulus was then added to either the perfusate or the bath and the perfusate was collected for another 30 min to measure the stimulated mucin release rate. Mucin in perfusates was quantified by periodic acid-Schiff's base dot-blot assay, using purified pig gastric mucin as a standard.

**Results:**

When applied luminally at 1 μM lubiprostone was ineffective at stimulating mucin release. When added to the serosal solution, 1 μM lubiprostone stimulated mucin release to ~300% of the unstimulated rate. As a positive control, serosal 1 μM prostaglandin E2 increased mucin release to ~400% of the unstimulated rate.

**Conclusions:**

These results support the idea that lubiprostone has prostaglandin-like actions on the intestine, which includes stimulation of mucin release. Stimulation of mucin release by lubiprostone may be protective in gastrointestinal conditions where loss of mucus is believed to contribute to pathogenesis. Thus, in addition to chronic constipation, there is greater potential for the therapeutic applications of lubiprostone.

## Background

Lubiprostone is a synthetic bicyclic fatty acid derivative of prostaglandin E1 used for chronic constipation. The best known action of lubiprostone is simulation of Cl- dependent fluid secretion. The originally proposed mechanism of lubiprostone is activation of the CLC2 chloride channel but this is controversial [1]. Recent work suggests that lubiprostone may act in a prostaglandin-like manner to stimulate cystic fibrosis transmembrane conductance regulator (CFTR) dependent Cl- and fluid secretion [2].

In the genetic disease cystic fibrosis (CF) there is impaired Cl^-^ and fluid secretion by affected epithelia contributing to the pathophysiology in this disease. Because lubiprostone can stimulate Cl^-^ secretion, we had previously investigated its use as a therapy for CF by testing its effects in a mouse model of CF, a *Cftr* knockout [3]. Treatment of CF mice with lubiprostone did ameliorate some of the CF related alterations. Lubiprostone treatment of CF mice accelerated gastric emptying, decreased small intestinal bacterial overgrowth, and reduced inflammation. These effects are independent of lubiprostone’s ability to stimulate Cl^-^ secretion because intestinal tissue from CF mice does not secrete Cl^-^ in response to lubiprostone [2]. Unexpectedly, in vivo administration of lubiprostone to CF mice resulted in greater mucus accumulation in the small intestine [3], mucus accumulation being one of the major phenotypes of CF. This effect on mucus accumulation is consistent with lubiprostone acting through a prostaglandin receptor, as prostaglandin E2 (PGE_2_) is a known potent stimulus for intestinal mucin release. Therefore, in this project we investigated the ability of lubiprostone to stimulate mouse intestinal mucin secretion, using an ex vivo organ bath approach.

## Methods

### Measurement of intestinal mucin release using an ex vivo system

**Figure 1:**
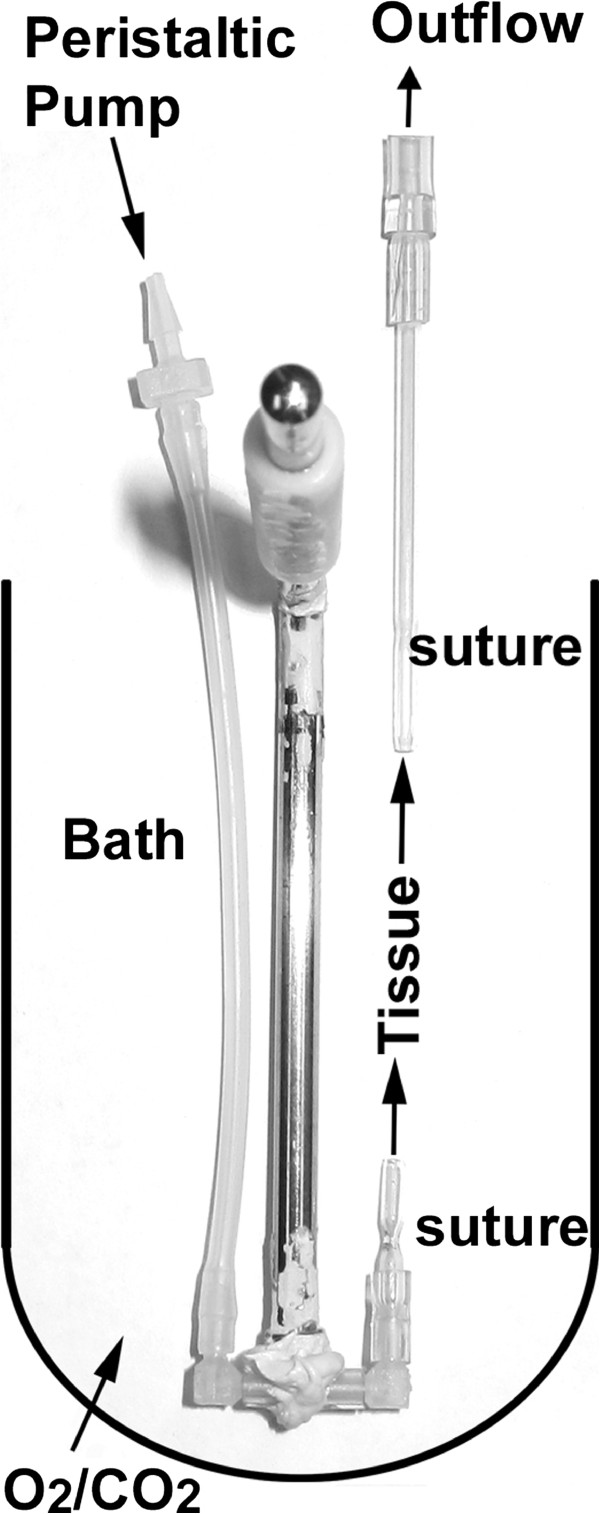
**Custom tissue holder for organ bath perfusion studies.** A stainless steel tissue holder was modified to hold a 4–5 cm segment of small intestine. The tissue was attached to the fire-polished glass capillaries with sutures at either end. The assembly was then placed in the 37°C thermostated organ bath which was filled with bicarbonate buffered Ringer’s solution and continuously gassed with 95% O_2_/5% CO_2_. The tissue was perfused with PBS using a peristaltic pump at a flow rate of ~0.2 mL min-1. The outflow was collected at 5 min intervals for the 1st 30 min (equilibration) followed by addition of stimulus and collection of outflow at 3 min intervals (stimulated)

Mucin released was measured similar to that described in [4]. Male C57BL/6 J mice (Jackson Labs, Bar Harbor, ME) were used at 10–12 weeks of age. All animal work was approved by the Institutional Animal Care and Use Committee of the University of Kansas Medical Center. Mice were killed by CO_2_ asphyxiation and cervical dislocation. The small intestine was removed into ice cold phosphate buffered saline (PBS) containing nifedipine (10^-6^ M) and indomethacin (10^-5^ M) to inhibit smooth muscle activity and endogenous prostaglandin production, respectively. After discarding the proximal most 4 cm of the small intestine, the next two adjacent segments of 4–5 cm of intestine (jejunum) were dissected from each mouse. The number of segments reported as (n) in the figure legends indicates the number of mice used. These segments were mounted by securing with thread at either end to fire-polished 1.2 mm diameter glass capillaries (see Figure 1). A stainless steel tissue holder (Biopac, Goleta, CA; catalog RXHOLDER-S) was modified to accept one of the glass capillaries by attaching a pair of plastic connectors (Cole-Parmer, Chicago, IL; kit #6365-90; 1.6 mm elbows) to one another by short pieces of tubing and epoxying them to the rod to make a U-shaped connector at the bottom (Figure 1). The other end of the U-shaped connector was attached to sylastic tubing through a peristaltic pump (Fisher Scientific, catalog 13-876-1) set to achieve a flow rate of ~0.2 mL min^-1^. The outflow tubing, attached to the upper glass capillary, was positioned about 1 cm above the upper level of the tissue to maintain a slight positive pressure in the segment to keep the lumen open. The lumen perfusate solution was PBS without glucose, warmed to 37°C. The mounted tissue was placed vertically in an organ bath (20 mL; Biopac). The bath was filled with bicarbonate buffered Ringer’s solution containing 10 mM glucose, continuously gassed with 95% O_2_/5% CO_2_, and thermostated to 37°C. Nifedipine (10^-6^) and indomethacin (10^-5^) were included in all solutions. The tissue was equilibrated under flow (~0.2 mL min^-1^) for 30 min. The perfusate collected during the final 10 min of the equilibration period was used to measure the unstimulated release rate. Stimulus was then added to either the perfusate solution or the bath solution, and the perfusate outflow was collected for another 30 min. The perfusate outflow during this latter period was used to measure the stimulated mucin release rate. 

### Quantification of released mucins

Mucin in perfusates was quantified by periodic acid-Schiff's base dot-blot assay, using a kit (Sigma; catalog 395B). Pig gastric mucin (Sigma, St. Louis, MO; catalog M1778) was used as a standard, prepared by suspension at 10 mg mL^-1^ in PBS using a probe-type sonicator. Serial 2-fold dilutions were prepared and in each assay 125 μg through 0.122 μg of mucin plus a blank were applied by vacuum filtration to nitrocellulose membrane in a dot-blot apparatus (Bio-Rad, Hercules, CA). A standard series of gastric mucins was included on every dot-blot. Perfusate outflow samples (50 μL) were applied in duplicate to the membrane as well. The membrane was removed from the apparatus and sequentially incubated in 3% acetic acid for 10 min, 6X diluted periodic acid for 15 min, 3% acetic acid washing for 10 min, 6X diluted Schiff’s Reagent for 10 min, 5 mg mL^-1^ sodium metabisulfite in water for 10 min, rinsing in water for 10 min, followed by air drying. The dried blots were scanned with a flatbed scanner (Hewlett Packard, Palo Alto, CA) and relative intensities measured using OptiQuant software (Kodak, Rochester, NY). The standard curve data were analyzed and sample unknowns determined using SigmaPlot software (Jandel Scientific, San Jose, CA; 'Standard Curves' feature in the 'Pharmacology' menu, with ‘Log X-scale’ and ‘Predict unknowns’). The PeakFit program (Jandel Scientific) was used to determine area under the curve (AUC) for 20–30 time points (unstimulated), and the 30–60 min time points (stimulated) which are presented as μg mucin min^-1^ g^-1^ tissue.

### Statistical analysis

Data are presented as means ± standard errors. When there are 2 groups of data, a *t*-test was used. When there are more than 2 groups an ANOVA with post-hoc Tukey’s test was used. P-values <0.05 are considered statistically significant.

## Results and discussion

**Figure 2:**
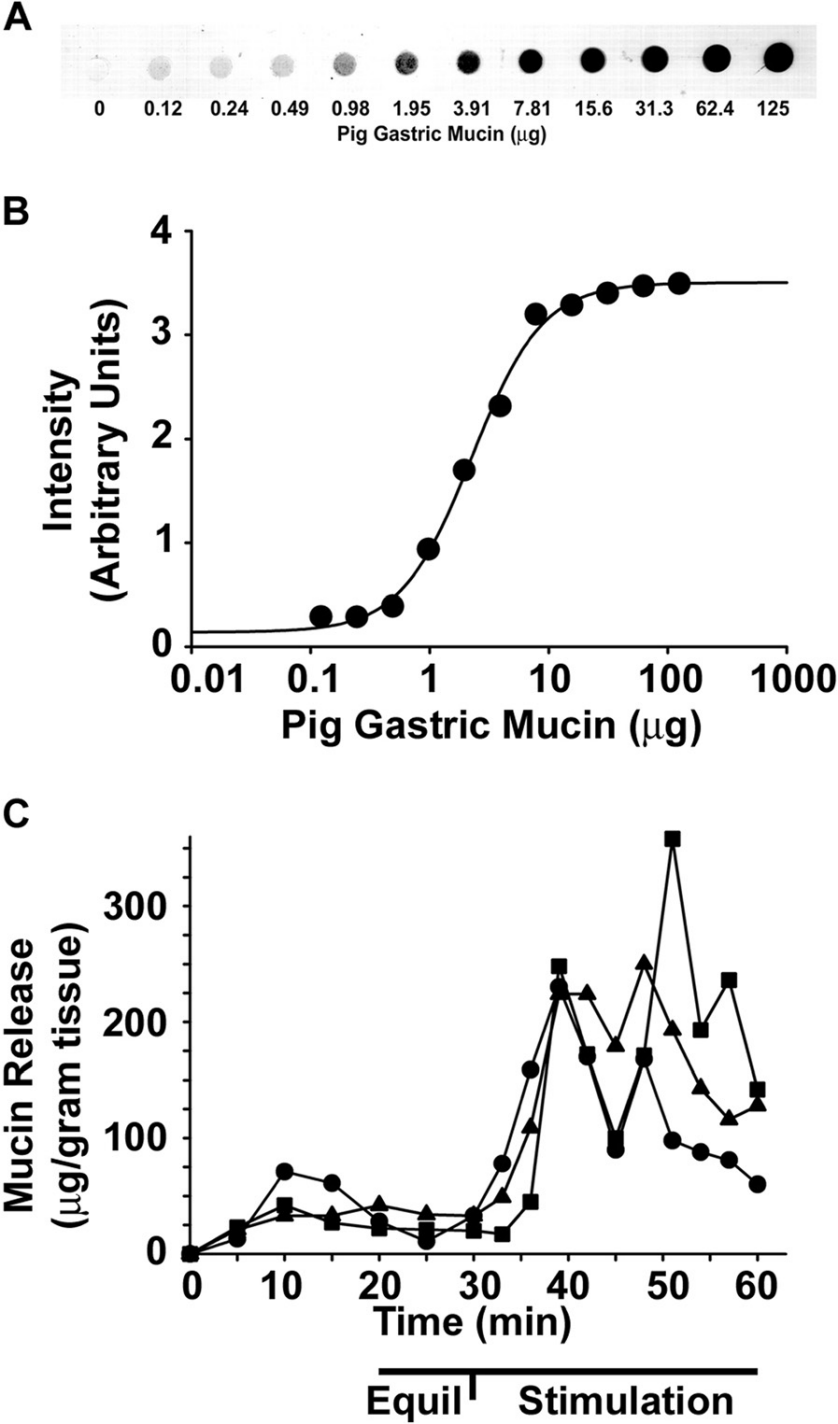
**Standard curve of pig gastric mucin and examples of PGE**_**2**_**-stimulated mucin release.** (**A**) Example of the dilution series of pig gastric mucin. Purified pig gastric mucin was prepared in PBS and 2-fold serial dilutions were made. Mucin was applied to nitrocellulose membrane in a dot-blot apparatus followed by periodic acid Schiff’s reaction. The dried blot was imaged on a flatbed scanner and analyzed as described in the Methods. (**B**) Standard curve generated using SigmaPlot software. (n = 13 series of standards; R^2^ = 0.997) (**C**) Examples of mucin release before and after addition of PGE_2_ (1 μM final) to the bath (serosal) solution. The 20–30 min data (Equil) were used to measure the area under the curve for the unstimulated period, and 30–60 min data (Stimulation) were used to measure the area under the curve for the stimulated period (Figure 2)

To measure mucin release from mouse small intestine, an ex vivo perfusion system was established similar to that in [4] using modifications as described in the Methods (Figure 1). It is assumed that with an adequate flow rate and the presence of bicarbonate ion in the perfusate and bath solutions, that the exocytosed mucins will expand and be mobile enough to be quantitatively included in the perfusate [4]. Mucin in perfusate outflow was quantified by periodic acid-Schiff's base dot-blot assay, using purified pig gastric mucin as a standard (see Methods). An example of the dot-blot of the dilution series of pig gastric mucin standards used is shown in Figure 2A. The intensity of a serial 2-fold dilution series of`mucin from 125 μg - 0.12 μg shows that mucin is detectable down to ~1 μg in a 50 μL aliquot of perfusate outflow (Figure 2A). When the data are analyzed using standard curve analysis software (SigmaPlot) the nonlinear regression indicates a very good fit (R^2^ = 0.997) (Figure 2B). 

**Figure 3:**
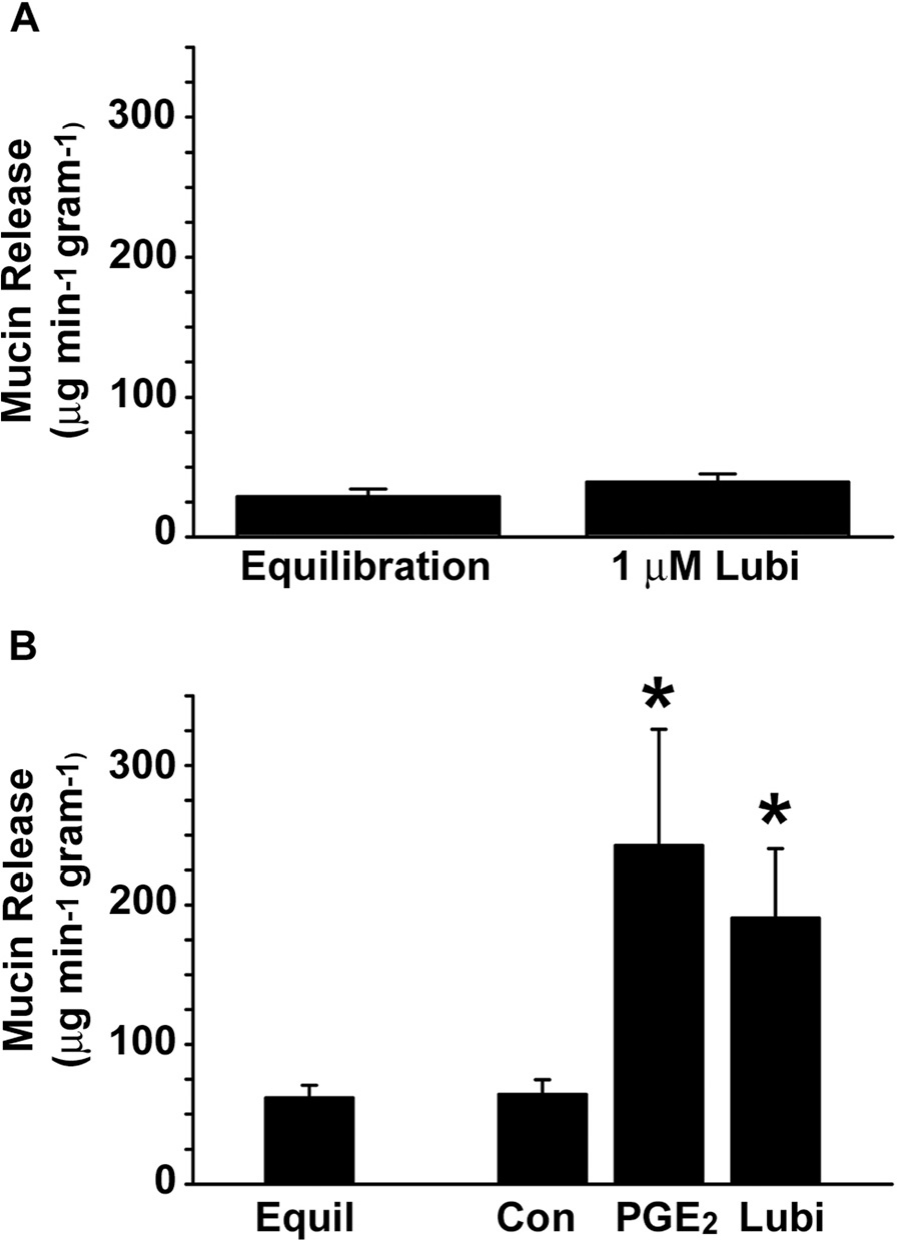
**Effect of lubiprostone on intestinal mucin release.** (**A**) Luminal Lubiprostone. Lubiprostone (1 μM final) was added to the perfusate (luminal) solution after a 30 min equilibration period. The areas under the curves for 20–30 min data (Equilibration) and 30–60 min data (1 μM Lubiprostone) were measured and divided by the time period to determine the rates of mucin release. (n = 2 intestinal segments) (**B**) Serosal PGE_2_, Lubiprostone. Vehicle (Control), PGE_2_ (1 μM final), or lubiprostone (Lubi, 1 μM final) was added to the bath (serosal) solution after a 30 min equilibration period. The areas under the curves for 20–30 min data (Equilibration; n = 26 intestinal segments) and 30–60 min data (Control unstimulated, n = 10; PGE_2_, n = 9; lubiprostone, n = 7) were measured and divided by the time period to determine the rates of mucin release. (*) p<0.05 vs Control

As a positive control, the known mucin secretagogue PGE_2_[4] was used. Examples from three independent intestinal preparations are shown in Figure 2C. There is variable mucin release during the equilibration period which becomes uniformly low during the final 10 min of equilibration. Upon addition of PGE_2_, there is an increase in mucin release whose timing is somewhat variable preparation-to-preparation (Figure 2C). Therefore, the area under the curve was calculated, divided by the 30 min stimulation period, and mucin release is expressed as μg mucin min^-1^ g^-1^ tissue, as shown in Figure 3. 

There is disagreement in the literature as to the membrane surface where lubiprostone acts. In some experiments, it was reported that lubiprostone acted when applied to the apical surface of epithelia [1,5] and in others when it was applied basolaterally [6]. Therefore, we tested application of lubiprostone to either surface. When applied to the luminal surface by addition to the perfusate solution, lubiprostone (1 μM) had no effect on mucin release (Figure 3A). Similar to luminal lubiprostone, luminal application of PGE_2_ (1 μM) also had no effect on mucin release (data not shown).

We next tested the effect on mucin release of lubiprostone added to the serosal surface (to the bath). For comparison, we used PGE_2_. As shown in Figure 3B, both PGE_2_ (1 μM) and lubiprostone (1 μM) stimulated mucin release. PGE_2_ increased the rate of mucin release to ~400% that of the unstimulated (equilibration) rate (Figure 3B). Lubiprostone also stimulated mucin release to over 300% that of the unstimulated rate (Figure 3B). The difference in stimulation of mucin release between PGE_2_ and lubiprostone was not statistically significant.

Because lubiprostone also stimulates bicarbonate-rich fluid secretion in the small intestine [7], it is likely that this effect of lubiprostone facilitates solubilization of exocytosed goblet cell mucin and its expansion to mucus. It has been shown that co-secretion of bicarbonate through a CFTR-dependent pathway is crucial for correct expansion of exocytosed mucins [4,8]. Since lubiprostone also activates CFTR-dependent bicarbonate secretion, mucin release and bicarbonate will be simultaneously stimulated by lubiprostone.

After this study was submitted, a report was published investigating the effects of lubiprostone stimulation in intestinal loop preparations of rat intestines and ex vivo human colonic samples [9]. They showed that lubiprostone caused a coordinated redistribution of several apical and basolateral ion transporters between cytoplasmic vesicles and the plasma membrane that collectively would support secretion at the same time as suppressing absorption, in villi of the small intestine and in colonic crypts. They also quantified mucin granule exocytosis by morphometric histological analyses and showed that lubiprostone caused depletion of goblet cells mucin granules in both small and large intestine. Our results that lubiprostone stimulates mucin release ex vivo from the mouse small intestine are in agreement with the results of this study.

## Conclusions

The results of this study support the idea that lubiprostone has prostaglandin-like actions on the intestine, which includes stimulation of mucin release. It remains to be determined which prostaglandin receptor(s) is/are activated by lubiprostone in the stimulation of mucin exocytosis by small intestinal goblet cells. It is known that mucin exocytosis and bicarbonate-rich fluid secretion are tightly coupled in the intestine [10] and that fluid secretion is impaired in EP3 PGE_2_ receptor knockout mice [11]. However, in human duodenum, fluid secretion can be stimulated by an EP4 selective agonist [12] and an EP4 antagonist blocks bicarbonate secretion in rat duodenum [7], making the EP4 receptor a likely candidate for mediating lubiprostone's effects on mucin release. Regardless of which prostaglandin receptor is involved, stimulation of mucin release by lubiprostone may be protective in gastrointestinal conditions where loss of mucus is believed to contribute to pathogenesis [13]. Thus, in addition to chronic constipation, there is greater potential for the therapeutic applications of lubiprostone.

## Abbreviations

CF: Cystic fibrosis; CFTR: Cystic fibrosis transmembrane conductance regulator; PBS: Phosphate buffered saline; PGE_2_: Prostaglandin E2.

## Competing interests

Support was provided by grant MSA-LUB-118 from Takeda Pharmaceuticals U.S.A., Inc. Takeda had no input into the design, execution, or data analysis. Takeda Pharmaceuticals is distributing the substance lubiprostone investigated in this study.

## Authors’ contributions

RCD performed the experiments, analyzed the data, and wrote the manuscript.

## Pre-publication history

The pre-publication history for this paper can be accessed here:

http://www.biomedcentral.com/1471-230X/12/156/prepub
